# The Piezo2 ion channel is mechanically activated by low-threshold positive pressure

**DOI:** 10.1038/s41598-019-42492-4

**Published:** 2019-04-23

**Authors:** Kyung Chul Shin, Hyun Ji Park, Jae Gon Kim, In Hwa Lee, Hawon Cho, Chanjae Park, Tae Sik Sung, Sang Don Koh, Sang Woong Park, Young Min Bae

**Affiliations:** 1 Department of Physiology, KU Open Innovation Center, Research Institute of Medical Science, Konkuk University School of Medicine, Chungju, Chungbuk 380-701 South Korea; 2 Sensory Research Center, Creative Research initiatives, College of Pharmacy, Seoul National University, Seoul, 151-742 South Korea; 3 Department of Physiology and Cell Biology, University of Nevada, Reno School of Medicine, Reno, NV 89557 USA; 4 Department of Emergency Medical Services, Eulji University, Seongnam, South Korea

**Keywords:** Ion channels in the nervous system, Neurophysiology

## Abstract

Recent parallel studies clearly indicated that Merkel cells and the mechanosensitive piezo2 ion channel play critical roles in the light-touch somatosensation. Moreover, piezo2 was suggested to be a light-touch sensing ion channel without a role in pain sensing in mammals. However, biophysical characteristics of piezo2, such as single channel conductance and sensitivities to various mechanical stimuli, are unclear, hampering a precise understanding of its role in touch sensation. Here, we describe the biophysical properties of piezo2 in human Merkel cell carcinoma (MCC)-13 cells; piezo2 is a low-threshold, positive pressure-specific, curvature-sensitive, mechanically activated cation channel with a single channel conductance of ~28.6 pS. Application of step indentations under the whole-cell mode of the patch-clamp technique, and positive pressures ≥5 mmHg under the cell-attached mode, activated piezo2 currents in MCC-13 and human embryonic kidney 293 T cells where piezo2 was overexpressed. By contrast, application of a negative pressure failed to activate piezo2 in these cells, whereas both positive and negative pressure activated piezo1 in a similar manner. Our results are the first to demonstrate single channel recordings of piezo2. We anticipate that our findings will be a starting point for a more sophisticated understanding of piezo2 roles in light-touch sensation.

## Introduction

Touch is one of cutaneous senses important for social interaction and sensing environmental changes. The role of Merkel cells in the sense of touch has been unclear for a long time since the discovery of the Merkel cell-neurite complex^1^. Nevertheless, recent parallel studies clearly indicated that Merkel cells and the mechanosensitive piezo2 ion channel expressed in Merkel cells and afferent Aβ fibers, play critical roles in the light-touch somatosensation^2–4^. Moreover, piezo2 was suggested to be a light-touch sensing ion channel without a role in pain sensing in mammals^5^. However, biophysical characteristics of piezo2, such as single channel conductance and sensitivities to various mechanical stimuli, are unclear, hampering a precise understanding of its role in touch sensation^6^. Poking or indentation of Merkel cells with a blunt glass pipette were reported to activate the piezo2 ion current under the whole-cell configuration of the patch-clamp technique^3–5^. However, application of negative pressure up to 190 mmHg failed to active piezo2 current under the cell-attached configuration^6^. By contrast, piezo1 ion channels could be activated both by poking and by negative pressure under the whole cell and cell-attached modes of patch-clamp, respectively^7^. It was suggested that piezo2 ion channels are expressed in the processes or spines of Merkel cells, where microelectrode access for cell-attached patch-clamp recording is not feasible^6^. However, the possibility that negative pressure is an inadequate stimulus for piezo2, and that piezo2 ion channels in Merkel cells are only sensitive to positive pressure (such as indentation or poking), has not been explored. Here, we describe the biophysical properties of piezo2 in human Merkel cell carcinoma (MCC)-13 cells; piezo2 is a low-threshold, positive pressure-specific, curvature-sensitive (CS), mechanically activated (MA) non-selective cation channel with a single channel conductance of ~28.6 pS. Our results are the first to demonstrate single channel recordings of piezo2 and its biophysical properties such as low-threshold mechanosensitivity and positive pressure preference. We anticipate that our findings will be a starting point for a more sophisticated understanding of piezo2 roles and suggest that, with its low-threshold, positive pressure-specific mechanosensitivity, piezo2 is an optimized and specialized sensor of light touch.

## Results

### The mechanically activated currents in human MCC-13 Merkel cells are piezo2-like and depend on positive pressure

**Figure 1:**
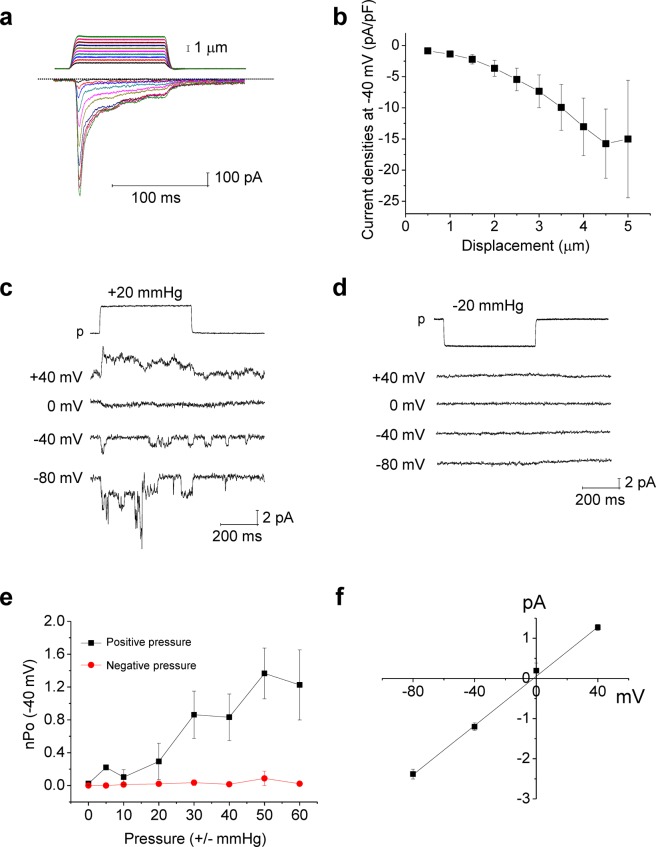
Recording of mechanically activated (MA) currents in human MCC-13 cells. **(a)** Representative traces of step indentation-induced whole-cell current (holding potential = −40 mV). **(b)** Summary of the current-stimulus (indentation) relationship (n = 6, except for 5 μm of displacement, where n = 3). (**c**) Representative traces of positive pressure-induced currents (cell-attached mode). (**d**) Representative traces showing no effect of negative pressure (cell-attached mode). (**e**) Summary of current (nPo)-pressure-relationships (n = 5–7 for positive pressure, n = 5–8 for negative pressure). (**f**) Single channel current (I)–voltage (V) plot of the positive-pressure induced currents, (n = 8–10). n, number of cells examined.

Step indentations under the whole-cell configuration of the patch-clamp technique evoked the mechanically activated (MA), time-dependent inward currents at a holding potential of −40 mV in MCC-13 cells (Fig. 1a,b), and the characteristics of these indentation-activated currents were qualitatively similar to those of piezo2 currents recorded in native Merkel cells^2–4^. The average capacitance of MCC-13 cells was 18.1 ± 1.7 pF (n = 19) in this study. The observed MA currents in MCC-13 cells were inactivated with a decay time constant of 10.4 ± 1.3 (n = 6, at −40 mV, fitted with single exponential decay function) during a 100 ms-long step indentation (Fig. 1a). The MA currents were inhibited by reduction of external Na^+^ or by external application of Gd^3+^ (50 μM) and ruthenium red (50 μM) (data not shown). These are consistent with the characteristics of previously reported piezo2 currents^2–4^. Further, we tried to record single channel currents of these indentation-activated currents in MCC-13 cells with the cell-attached mode of the patch-clamp technique. Interestingly, positive pressure application activated the mechanosensitive currents, whereas application of negative pressure failed to activate currents (Fig. 1c,d). The open probability (nPo)-pressure relationships of these MA currents with positive and negative pressure are summarized in Fig. 1e. Single channel current (I)-voltage (V) relationship of the positive pressure-activated current (representative trace of which is shown in Fig. 1c) is summarized in Fig. 1f. The single channel conductance of the positive pressure-activated currents was ~28.6 pS and the reversal potential (E_rev_) was −1.8 mV. These results show that positive but not negative pressure can activate a mechanosensitive, non-selective cation current in human MCC-13 Merkel cells, whose single channel conductance is similar to that of mammalian piezos, such as piezo1 and piezo2^6–8^. Single channel conductance of piezo1 was reported to be ~23 and ~30 pS under similar experimental conditions^7,8^. Although single channel conductance of piezo2 is currently unclear due to a lack of reported single channel recordings of piezo2, it was expected to be ~32.5 pS as inferred from stationary noise analysis of the whole-cell currents in rat Merkel cells^6^. The sensitivity of the channels in a patch to positive but not negative pressure suggests that the observed Piezo2-like currents in MCC-13 cells are CS current because the positive pressure-specificity means that the channels are not driven by membrane tension since the tension is independent of sign of the pressure gradient. This sign dependence suggests that the channels are responding to changes in membrane curvature, not tension. Under positive pressure the patch folds back on the seal creating a region of high curvature at the periphery of the patch^9–11^.

### Piezo 1 currents are activated by both positive and negative pressure

**Figure 2:**
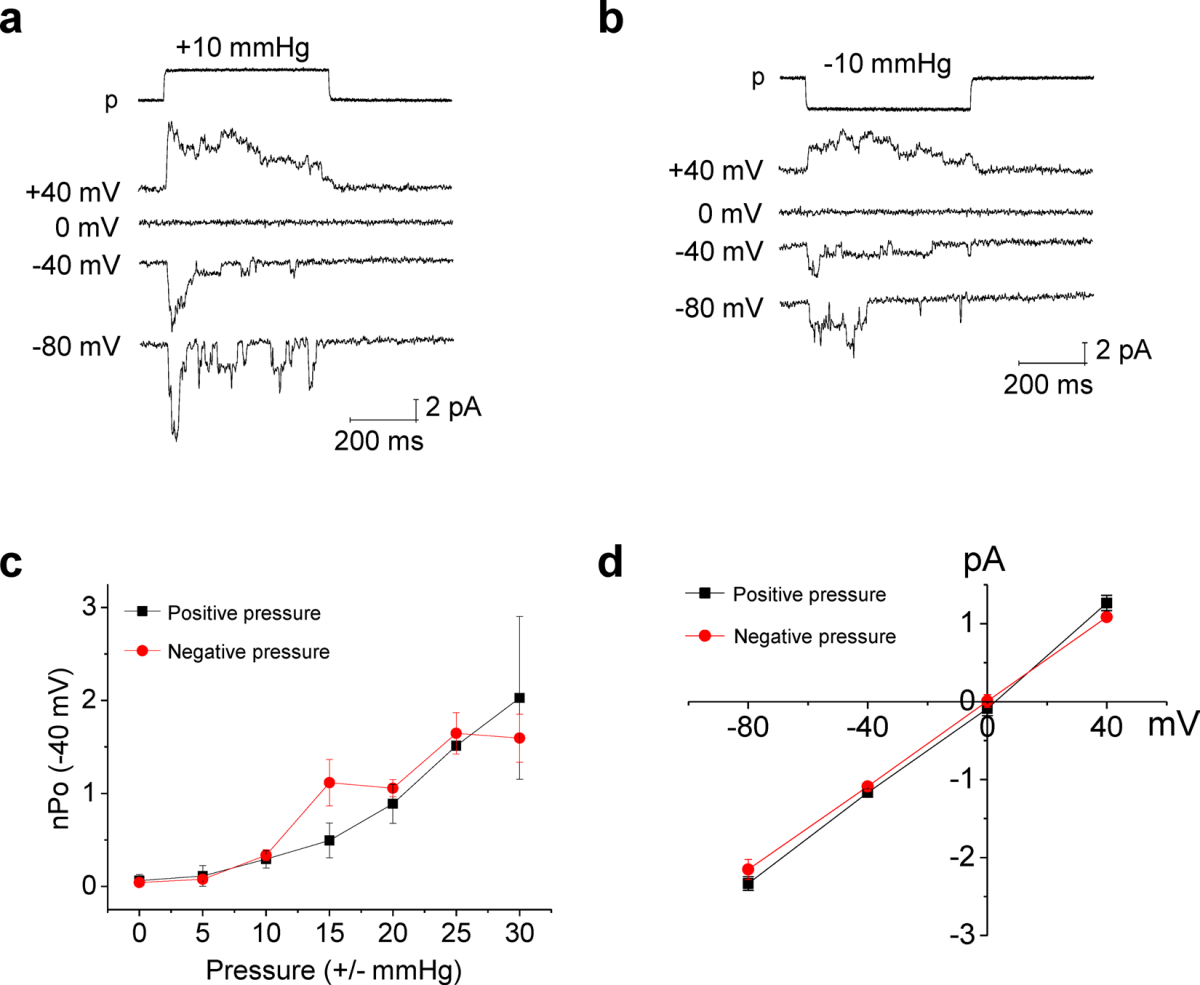
Recordings of MA currents in neuro2A (N2A) cells. (**a**,**b**) Representative traces of positive and negative pressure-induced currents at various membrane potentials under cell-attached patch mode. (**c**) Summary of current (nPo)-pressure (negative & positive)-relationships in N2A cells (n = 8 for positive pressure, n = 9 for negative pressure). (**d**) Single channel I-V relationships of positive and negative pressure-induced currents (n = 5 for positive pressure, n = 6–9 for negative pressure). n, number of cells examined.

**Figure 3:**
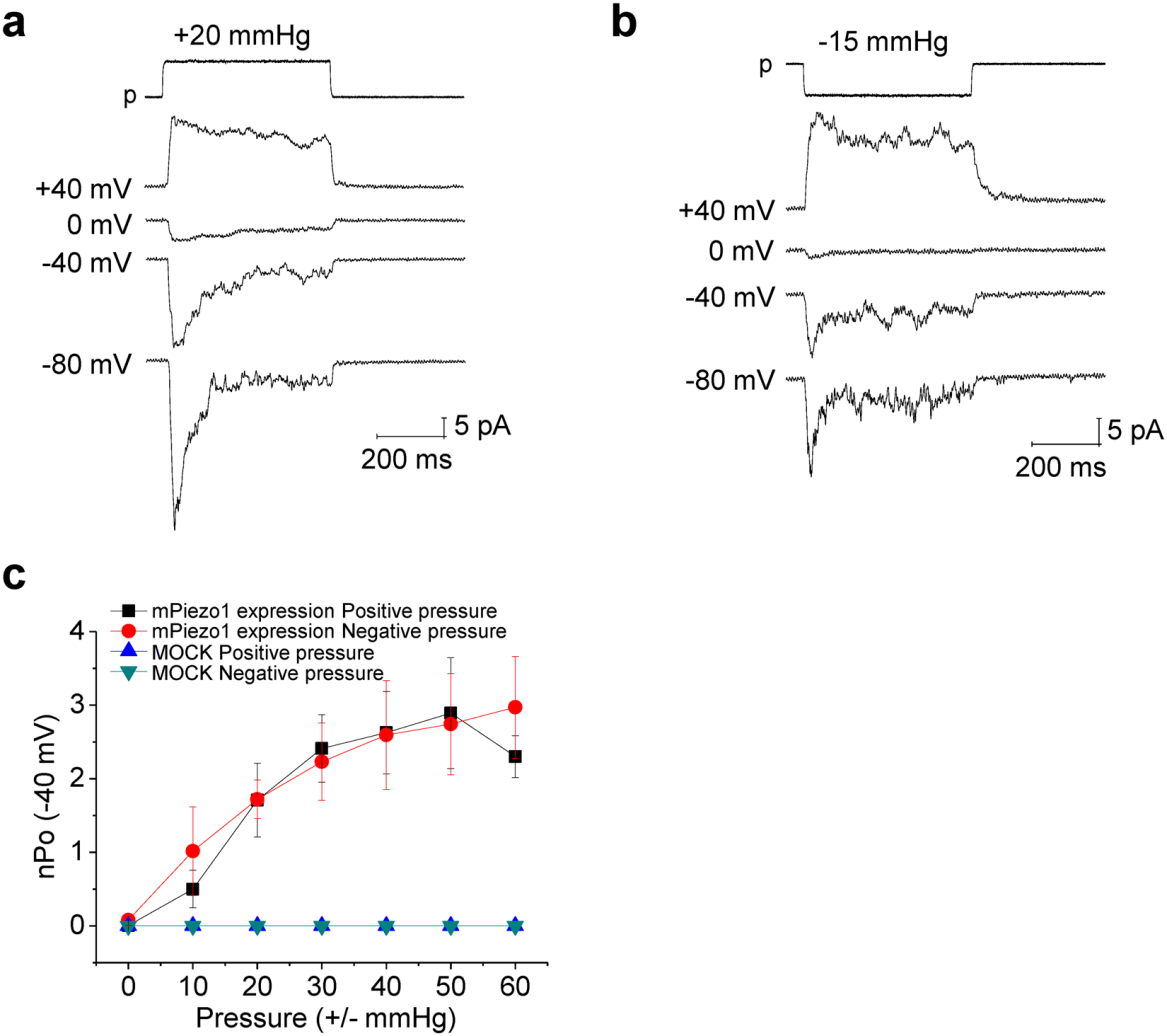
Effects of positive and negative pressure on transfected mpiezo1 channels in HEK293T cells. **(a)** Representative traces of positive pressure-induced currents (cell-attached mode). (**b**) Representative traces of negative pressure-induced currents (cell-attached mode). **(c)** Current (nPo)-pressure relationships of the mpiezo1 channels expressed in HEK293T cells (at −40 mV). n = 9 for mpiezo1 expression groups, n = 13–14 for controls (mock). n, number of cells examined.

In order to verify that the positive pressure-specific, curvature-sensitive activation of the piezo2-like current in MCC-13 cell was a genuine response and not an unspecific artifact, we performed similar experiments in Neuro2A (N2A) cells, where piezo1 was reportedly expressed^7^, and the piezo1 current found to be activated by both indentation and negative pressure^7^. Accordingly, both positive and negative pressure activated the pressure-dependent currents to a similar extent (Fig. 2a,b). The nPo-pressure relationships of the positive and negative pressure-activated currents (Fig. 2c) indicate that the piezo1 current in N2A cells is activated similarly by positive and negative pressure. The single channel conductance of these mechanically activated currents was 26.9 ± 2.1 pS (positive pressure) and 27.1 ± 0.7 pS (negative pressure), and the E_rev_ was +0.9 mV (positive pressure) and 0 mV (negative pressure) (Fig. 2d). These biophysical properties are very similar to those previously reported for the mammalian piezo1 channels^7,8^. Under heterologous overexpression system, the piezo1 channels were also similarly activated by both positive and negative pressure (Fig. 3). Figure 3a,b shows representative cell-attached patch clamp recording of mouse Piezo1 currents, of which channels were overexpressed in HEK293T cells, by positive and negative pressure, respectively. Taken together, the results in Figs 1–3 support the hypothesis that the piezo2-like MA currents in MCC-13 cells are CS currents specifically activated by positive pressure, whereas piezo1 currents are stretch sensitive currents, which are non-specifically activated by both positive and negative pressure.

### Expression of piezo2 in MCC-13 cells

Next, using immunocytochemistry and western blotting, we examined whether MCC-13 cells express piezo2 ion channels. Expression of piezo2 in MCC-13 cells was clearly confirmed with immunocytochemistry and western blotting (Supplementary Data Fig. 1), whereas no piezo2 was detected in N2A cells (Supplementary Data Fig. 1a). HEK293T cells and bladder tissue were used as negative and positive controls of piezo2 expression, respectively (Supplementary Data Fig. 1b)^7^.

### The positive pressure-activated currents in MCC-13 cells are piezo2-dependent

**Figure 4:**
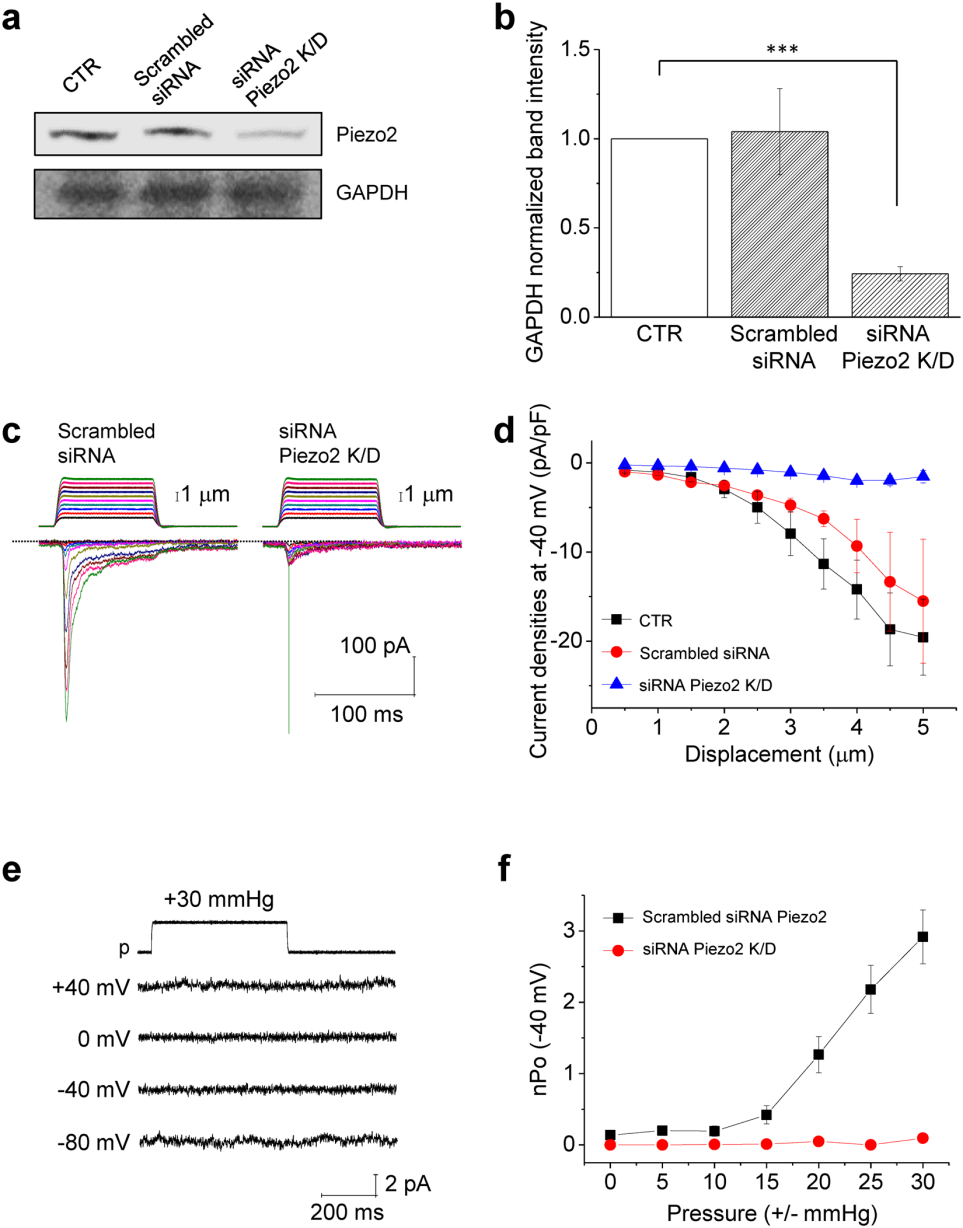
Effects of piezo2 channel knockdown on activation of MA currents in MCC-13 cells. **(a)** A representative western blot of piezo2 in control, scrambled siRNA, and piezo2 siRNA. (**b**) Bar graph summarizing piezo2 knockdown shown in panel (a) (n = 3). ***indicates *p* < 0.001 vs control or scrambled siRNA. **(c)** Representative traces of step indentation-induced currents (holding potential = −40 mV) in scrambled siRNA (left), and piezo2 knockdown (right) MCC-13 cells. (**d**) Comparison of the current-stimulus (indentation) relationship between control (n = 16, except for displacements of 4, 4.5, and 5 μm, where n = 15, 14, and 13, respectively), scrambled siRNA (n = 10–12, except for displacements of 4.5 and 5 μm, where n = 7 and 4, respectively) and piezo2 knockdown MCC-13 cells (n = 13, except for displacements of 4, 4.5, and 5 μm, where n = 11, 5, and 2, respectively). **(e)** Representative cell-attached traces showing no effect of positive pressure in the piezo2 knockdown MCC-13 cells. **(f)** Summary of the current (nPo)-pressure relationships in the piezo2 siRNA-treated MCC-13 cells (n = 14–23), n = 16–22 for scrambled siRNA Piezo2 group. n, number of cells examined.

In order to confirm that the positive pressure-activated, CS currents in MCC-13 cells were an actual piezo2-dependent current, we examined the effect of piezo2 knockdown on the positive pressure-activated currents in MCC-13 cells. Figure 4a and b show that the piezo2 siRNA successfully knocked down piezo2. In piezo2 knockdown MCC-13 cells, the MA inward currents induced by step indentations were greatly attenuated (Fig. 4c). The comparison of the current-indentation relationship (Fig. 4d) clearly shows that the indentation-dependent whole-cell current in MCC-13 cells is conducted through piezo2 channels. Accordingly, application of positive pressure up to 30 mmHg failed to activate MA currents in MCC-13 cells treated with piezo2 siRNA (Fig. 4e–f).

### The positive pressure-specificity of piezo2 is not cell-specific

**Figure 5:**
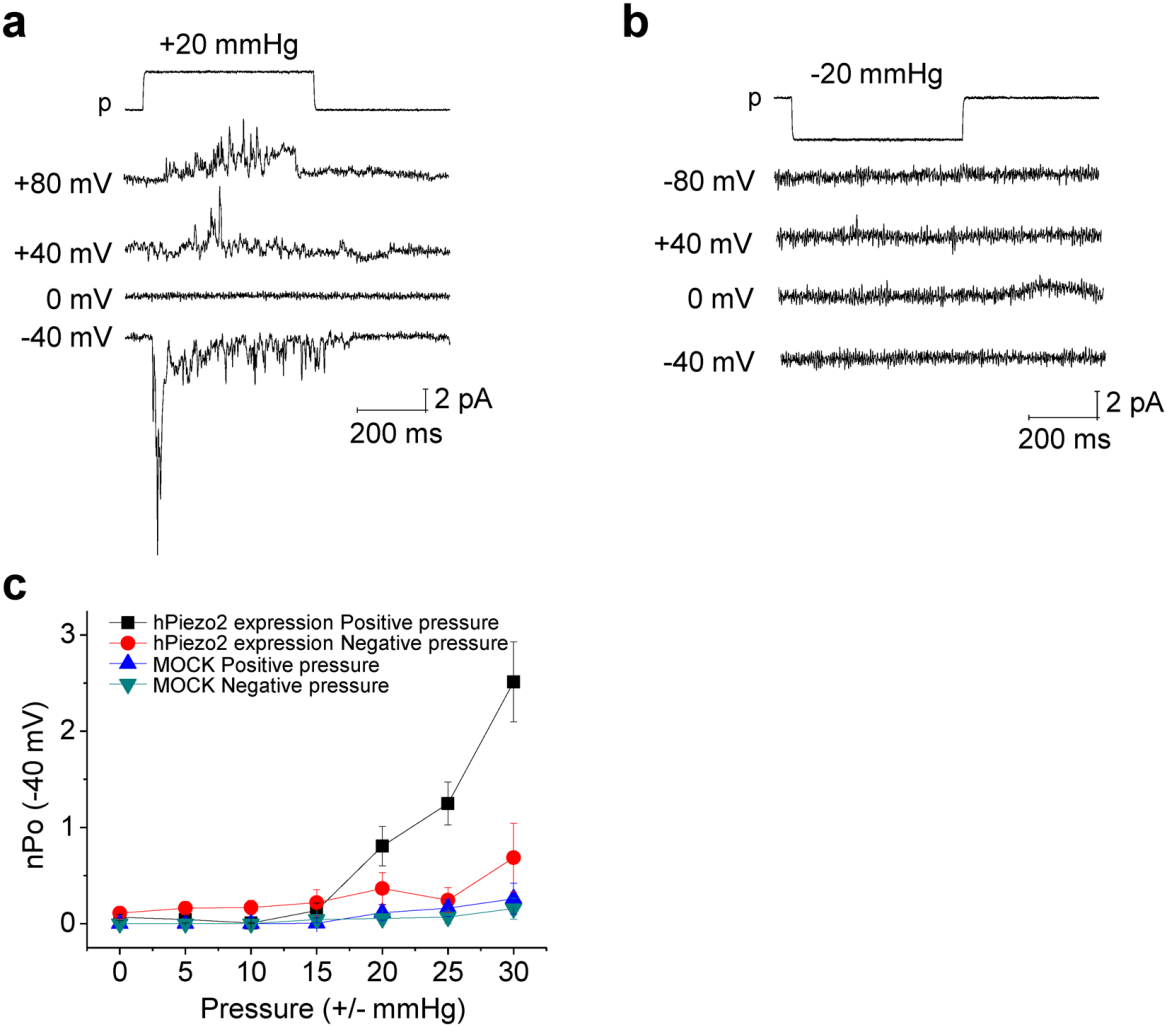
Effects of positive and negative pressure on transfected hpiezo2 channels in HEK293T cells. **(a)** Representative traces of positive pressure-induced currents (cell-attached mode). **(b)** Representative traces showing no effect of negative pressure (cell-attached mode). **(c)** Current (nPo)-pressure relationships of the hpiezo2 channels expressed in HEK293T cells (at −40 mV). n = 10–12 for hpiezo2 expression groups, n = 13–14 for controls (mock). n, number of cells examined.

Finally, we examined the effect of positive and negative pressure on piezo2 ion channels overexpressed in HEK293T cells. Human Piezo2 channels overexpressed in HEK293T cells also showed positive pressure-specific activation with little negative pressure-sensitivity (Fig. 5a–c), indicating that the positive pressure-specific mechanosensitivity is not cell-specific but from local curvature of cell membrane in which the Piezo2 channels are present. The curvature sensitivity of piezo2 would be relevant when the piezo2 functions as a light-touch sensor in the spines of Merkel cells^6^.

## Discussion

In the present study, we demonstrated that i) the human MCC-13 Merkel cells express mechanosensitive piezo2 channels, and that ii) piezo2 is a positive pressure-specific MA ion channel without negative pressure sensitivity. In contrast to piezo2, piezo1 was nonspecifically activated by both positive and negative pressure, as previously indicated^7,8^. These observations indicate that the Piezo2 channels sense membrane curvature rather than membrane tensions, whereas Piezo1 channels sense the membrane tension. In fact, similar CS channels with comparable single channel conductance (~50 pS) were previously reported in astrocytes^12^. Interestingly, astrocytes are relatively with higher expression of piezo2 channels than that of piezo1^13^. The functional expression of the CS-sensitive piezo2 and its clinical roles in astrocytes warrant further investigation.

A recent study by Ikeda *et al*.,^6^ attempted to record a single piezo2 channel current in intact Merkel cells of rat whisker hair follicle, after they^3^ and others^2,4,5^ first reported that piezo2 critically contributed to the light-touch somatosensation in Merkel cells and their afferent Aβ fibers. However, Ikeda *et al*.,^6^ failed to record the single channel current of piezo2 in intact Merkel cells. They concluded that piezo2 localization domains are preferentially present in spines of Merkel cells, and that the patch-pipette under the cell-attached mode could not reach the localization domains in the spines. Their conclusion may be correct and the localization domain of piezo2 in the MCC-13 cells used in this study may differ from that of intact rat hair follicle Merkel cells. However, another hypothesis, namely, that negative pressure is not an adequate stimulus for activating piezo2, is also possible. In this study, we clearly demonstrated that piezo2 is a positive pressure-specific MA channel, indicating that piezo2 is a CS channel rather than stretch-sensitive channel. In this regard, negative pressure and probably hypotonic swelling, usually applied for recording MA channels, would be unable to activate piezo2 channels. Moreover, piezo2 had a very low activation threshold, with only 5–10 mmHg of positive pressure sufficient for activation. Considering the recent estimation of tensile force by positive and negative pressure in the membrane patches under cell-attached patch pipettes^14^, the threshold would be even lower than the 5 mmHg of positive pressure. From these, we suggest that piezo2 is a low-threshold positive pressure-specific MA ion channel and that these properties would make piezo2 a specialized light-touch sensing channel in Merkel cells.

The positive pressure-specificity of the mechanically activated piezo2 channel is not an artifact related to a seal-break between the patch pipette and the cell membrane, based on the following observations: i) the positive pressure-specificity was found only for piezo2, whereas piezo1 was activated by both negative and positive pressure under a similar experimental setting, ii) treatment with siRNA against piezo2 in MCC-13 cells clearly suppressed the positive pressure-activation of piezo2 current, and iii) genetically overexpressed piezo2 in HEK293T cells also showed positive pressure-specific activation, whereas control (mock-treated) HEK293T cells had little MA current in response to positive or negative pressure. The mechanism of how piezo2 is specifically activated by positive pressure (i.e., curvature sensitivity) is currently unknown. Future mechanistic studies are required for elucidating the positive pressure specificity or the curvature sensitivity of piezo2.

In this study, about 50% of MCC-13 cells showed the CS piezo2 currents in response to the positive-pressure under cell-attached recordings, and the remaining half did not show any current in response to either negative or positive pressure up to 60 mmHg. This is in contrast with the observation where most MCC-13 cells showed piezo2 currents in response to the indentation stimuli under whole-cell recordings. The impression is that although we used the MCC-13 cell line, multiple factors including culture condition and way of cell isolation affect the functionality of CS piezo2 currents in MCC-13 cells. Notably, the previously reported CS current in astrocytes seems to have faced the same problem^12^. Permissive factors for the activation of CS piezo2 current warrant further studies.

In conclusion, we report for the first time a single channel recording of the piezo2 MA ion current, and the biophysical properties of piezo2, which point to a role for piezo2 in light-touch somatosensation in Merkel cells or its afferent nerve fibers.

## Methods

### Electrophysiology

Mechanically activated currents were recorded under the conventional whole-cell configuration and under the cell-attached configuration of the patch-clamp technique. We used an Axopatch 200B amplifier and P-clamp10 software (Axon Instruments, Sunnyvale, CA, USA) for recording ion currents. The ion currents were sampled with 10 kHz frequency after low-pass filtered at 1 kHz. For conventional whole-cell current recording, we used normal Tyrode (NT) bath solution, composed of: 143 mM NaCl, 5.4 mM KCl, 0.33 mM NaH_2_PO_4_, 0.5 mM MgCl_2_, 5 mM HEPES, 1.8 mM CaCl_2_, and 11 mM glucose (pH 7.4 with NaOH), and pipette solution, composed of: 133 mM CsCl, 4 mM MgATP, 10 mM HEPES, 5 mM EGTA, 1 mM CaCl_2_, 1 mM MgCl_2_, 0.4 mM Na_2_GTP adjusted to pH 7.3 with CsOH. For cell-attached patch-clamp recordings, we used patch pipettes with a resistance of 1–2 MΩ and filled with pipette solution composed of 143 mM NaCl, 5.4 mM KCl, 0.33 mM NaH_2_PO_4_, 5 mM HEPES, 0.5 mM MgCl_2_, 1.8 mM CaCl_2_, 11 mM glucose (pH 7.4 with NaOH), and a bathing solution consisting of: 140 mM KCl, 8.4 mM NaCl, 10 mM HEPES, 0.5 mM MgCl_2_, 11 mM glucose (pH 7.4 with KOH). Numbers (n) in the results indicate the numbers of cells examined. Single channel current amplitudes were determined after a Gaussian fitting analysis (Origin 8.0 or 9.0 software) of the full trace histograms of cell-attached recordings.

### Mechanical Stimulation

For mechanical stimulation during whole-cell current recording, step indentations were applied using a fire-polished glass pipette (tip diameter 2–4 μm), positioned at an angle of approximately 70° to the surface of the cell. The pipette was controlled by a piezo controller and amplifier (NV 40/1, piezo system, Jena, Germany). A series of mechanical steps in 0.5 μm increments was applied every 10 s, and the stimulus was applied for 100 ms.

In cell-attached single channel recordings, positive or negative pressure was applied via patch pipettes using a high speed pressure clamp system (HSPC-1; ALA-scientific, Farmingdale, NY, USA). Positive and negative pressure step pulses were applied in 5 mmHg increments at every 10 s and the pressure was applied for 500 ms.

### Cell culture

MCC-13 cells were grown in RPMI 1640 medium (GE healthcare, ﻿Pittsburgh, Pennsylvania, USA) and Neuro2A (N2A) and HEK293T cell lines were cultured in DMEM (Dulbecco’s Modified Eagle’s Medium; GE healthcare, ﻿Pittsburgh, Pennsylvania, USA). RPMI 1640 medium contained 15% fetal calf serum and 1% penicillin-streptomycin. DMEM medium contained 10% fetal bovine serum and 1% penicillin-streptomycin. For electrophysiology recordings, cells were placed in the patch-clamp chamber after treatment with 10% trypsin-EDTA for 3 min.

### Transfection protocols

For mPiezo1 and hPiezo2 transfections, HEK293T cells were grown to 80% confluence in a 60-mm dish. mPiezo1-IRES-eGFP was a gift from Ardem Patapoutian (Addgene plasmid # 80925; http://n2t.net/addgene:80925; RRID:Addgene_80925). hPiezo2 cDNA was a gift from Uhtaek Oh (Korea institute of science and technology, Seoul, Korea). The experiment was performed according to Thermo’s transfection protocol. The culture medium was replaced with 5.4 ml fresh medium containing 10% fetal bovine serum (FBS). Media (600 μl Opti-MEM medium and 12 μl turbofect transfection reagent and 6 μg of hPiezo2 DNA or mPiezo1 DNA) were mixed and incubated for 20 min at ~25 °C. After incubation, the mixed media with the DNA-lipid complex were added to the 60-mm dish containing the cultured cells. Cells were used after 2 days.

For small interfering RNA (siRNA) transfections, MCC-13 cells were grown to 80% confluence in a 60-mm dish and starved in RPMI 1640 without FCS for 12 h. The culture medium was replaced with 1.6 ml fresh medium with 15% FCS. Media1 [198 μl RPMI 1640 and 2 μl of 25 μM Piezo2 siRNA (L-013925-02-0010, Thermo Scientific, Waltham, Massachusetts, USA)] and Media2 (193 μl RPMI 1640 and 7 μl DharmaFECT) were incubated at ~25 °C for 5 min. After incubation, media1 and media2 were mixed and incubated for 30 min at ~25 °C. Then, 400 μl of the mixed media were added to the 60-mm dish containing the cells. Cells were used after 2 days.

### Western blotting

MCC-13 and HEK293T cells were grown to 80% confluence, and starved in medium without FCS or FBS for 12 h. Bladder tissue was isolated from 10 week-old Sprague Dawley rats. The experiments were conducted in accordance with the National Institutes of Health guidelines for the care and use of animals, and the institutional animal care and use committee of Konkuk University approved this study. Whole bladder tissues were homogenized and snap-frozen in liquid nitrogen. For western blotting, bladder tissue samples and MCC-13 and HEK293T cells were lysed in RIPA buffer (TNT research LTD KOREA). Samples were centrifuged at 14,000 *g* for 15 minutes at 4 °C, and the supernatant was collected. The samples were run on an 8% SDS-polyacrylamide non-reducing gel and then transferred to a PVDF membrane (Millipore, Bedford, MA, USA). Rabbit primary antibodies against Piezo2 (1:500; Abcam, Cambridge, MA, USA) and secondary antibodies (1:1000; Cell Signaling Technology, Danvers, MA, USA) were used in the western blot. Signals were visualized by Las-4000 (Fujifilm, Tokyo, Japan).

### Immunocytochemistry

Neuro2A cells and MCC-13 cells were grown to 80% confluence in the Labtek chamber slide system (Thermo Scientific, Waltham, Massachusetts, USA). The cells were treated with 4% paraformaldehyde for 30 min, 0.1% Triton X-100 for 10 min, and 5% BSA blocking solution for 1 h. The cells were then incubated overnight at 4 °C with rabbit primary antibodies against piezo2 (1:500, Abcam, Cambridge, MA, USA). Secondary antibodies conjugated to Cy2 were used (1:1000; Alomone Labs, Jerusalem, Israel). DNA was stained for 1 h with TOPRO-3 (1:1000, Life Technologies). Cells were observed under a confocal microscope (Zeiss LMS-710).

### Data analysis

Origin 8.0 or 9.0 software (Microcal Software, Inc., Northampton, MA, USA) was used for data analysis. The results are shown as means ± SEM. Paired or independent Student’s *t*-tests were used to test for significance as appropriate and *p* < 0.05 was regarded as significant.

## Supplementary information

SI of Piezo2 is a low-threshold, positive pressure-specific mechanically activated ion channel
