# Supplementary material for: The Piezo2 ion channel is mechanically activated by low-threshold positive pressure
Source: Sci Rep. 2019 Apr 23;9:6446. doi: 10.1038/s41598-019-42492-4 (PMC6478859; doi:10.1038/s41598-019-42492-4)
Supplement: Supplementary file 1 — SI of Piezo2 is a low-threshold, positive pressure-specific mechanically activated ion channel [file 41598_2019_42492_MOESM1_ESM.pdf]

Supplementary Information

Piezo2 is a low-threshold, positive pressure-specific mechanically activated ion channel

Kyung Chul Shin[1*], Hyun Ji Park[1*], Jae Gon Kim[1], In Hwa Lee[1], Hawon Cho[2], Chanjae Park[3], Tae Sik Sung[3], Sang Don Koh[3], Sang Woong Park[4], Young Min Bae[1]

[1]Department of Physiology, KU Open Innovation Center, Research Institute of Medical Science, Konkuk University School of Medicine, Chungju, Chungbuk 380-701, South Korea

[2]Sensory Research Center, Creative Research initiatives, College of Pharmacy, Seoul National University, Seoul 151-742, South Korea

[3]Department of Physiology and Cell Biology, University of Nevada, Reno School of Medicine, Reno, NV, USA 89557

[4]Department of Emergency Medical Services, Eulji University, Seongnam, South Korea.

Correspondence and requests for materials should be addressed to Y.M.B. (ymbae30@kku.ac.kr) or to S.W.P (swpark@eulji.ac.kr).

*K.C.S. and H.J.P. contributed equally to this work.

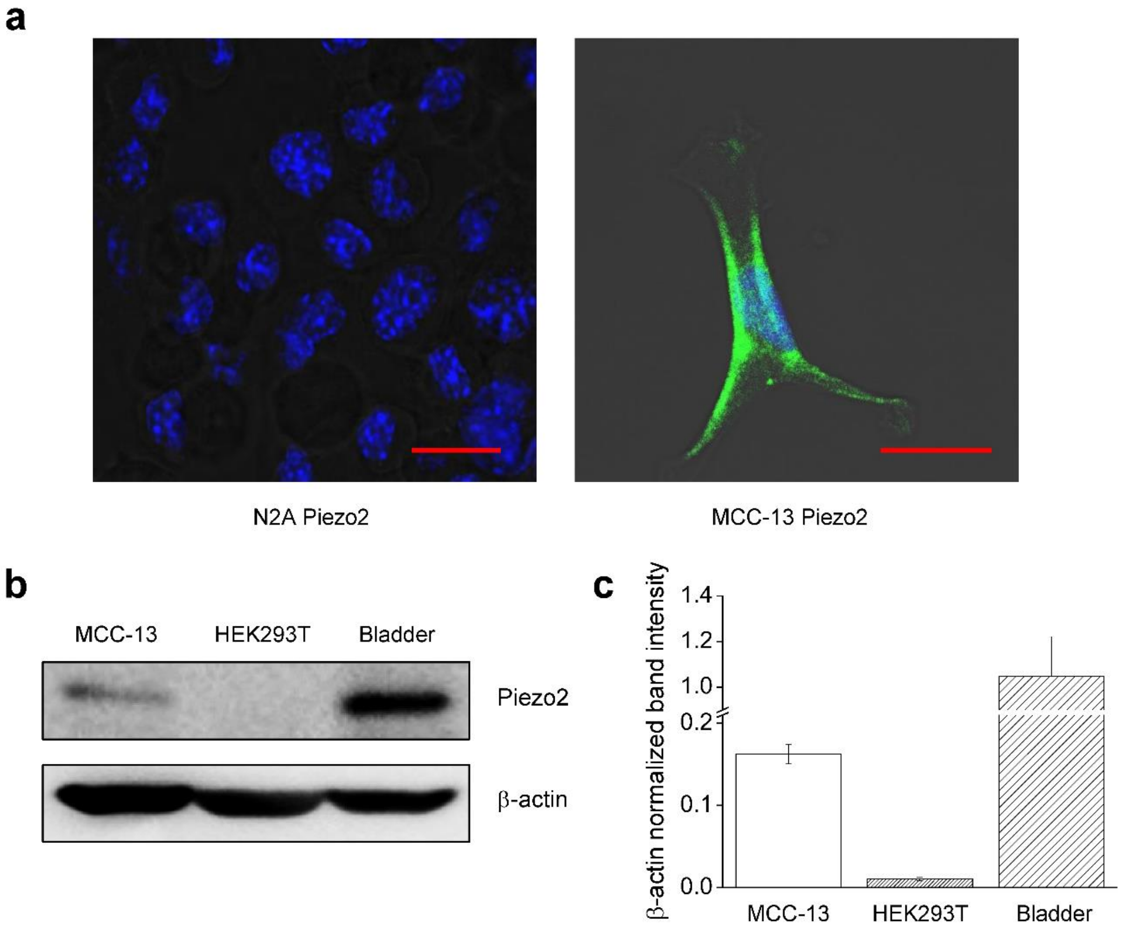

Figure S1. Expression of piezo2 channel in the MCC-13 cell line. **a,** Representative immunocytochemistry results for piezo2 expression in N2A (left panel) and MCC-13 (right panel) cells. Blue color indicates nucleic acid and green color piezo2 channels. Scale bar = 20 μm. **b,** A representative western blotting result of piezo2 expression in MCC-13, HEK293T (negative control), and bladder tissue (positive control). **c,** Quantification of panel **b** (n = 3).

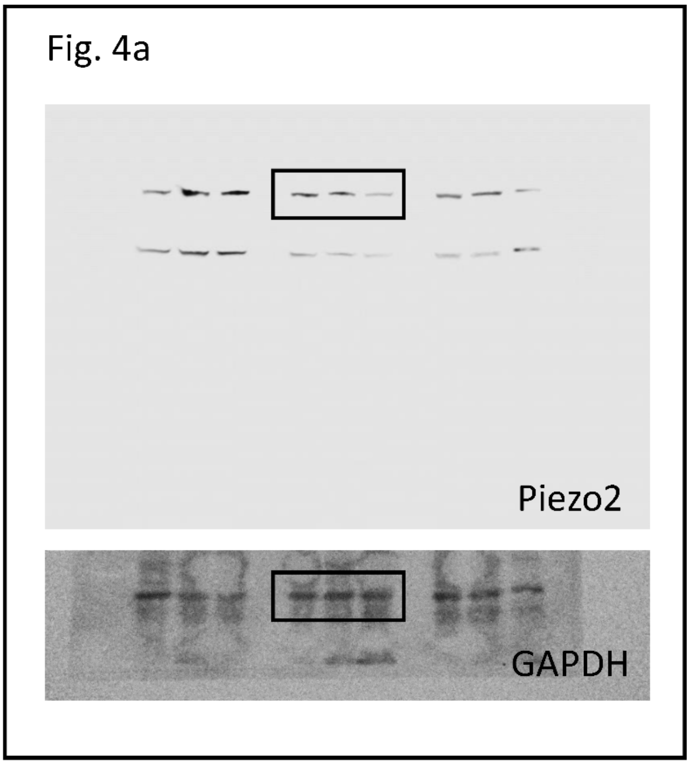

Figure S2. Uncropped Western blot for the data in Figure 4.
